# Role of Insulin-Like Growth Factor Binding Protein-3 in 1, 25-Dihydroxyvitamin-D_**3**_-Induced Breast Cancer Cell Apoptosis

**DOI:** 10.1155/2013/960378

**Published:** 2013-04-18

**Authors:** C. Brosseau, G. Pirianov, K. W. Colston

**Affiliations:** Division of Clinical Sciences, St George's University of London, London SW17 0RE, UK

## Abstract

Insulin-like growth factor I (IGF-I) is implicated in breast cancer development and 1, 25-dihydroxyvitamin D_3_ (1, 25-D_3_) has been shown to attenuate prosurvival effects of IGF-I on breast cancer cells. In this study the role of IGF binding protein-3 (IGFBP-3) in 1, 25-D_3_-induced apoptosis was investigated using parental MCF-7 breast cancer cells and MCF-7/VD^R^ cells, which are resistant to the growth inhibitory effects of 1, 25-D_3_. Treatment with 1, 25-D_3_ increased IGFBP-3 mRNA expression in both cell lines but increases in intracellular IGFBP-3 protein and its secretion were observed only in MCF-7. 1, 25-D_3_-induced apoptosis was not associated with activation of any caspase but PARP-1 cleavage was detected in parental cells. IGFBP-3 treatment alone produced cleavage of caspases 7, 8, and 9 and PARP-1 in MCF-7 cells. IGFBP-3 failed to activate caspases in MCF-7/VD^R^ cells; however PARP-1 cleavage was detected. 1, 25-D_3_ treatment inhibited IGF-I/Akt survival signalling in MCF-7 but not in MCF-7/VD^R^ cells. In contrast, IGFBP-3 treatment was effective in inhibiting IGF-I/Akt pathways in both breast cancer lines. These results suggest a role for IGFBP-3 in 1, 25-D_3_ apoptotic signalling and that impaired secretion of IGFBP-3 may be involved in acquired resistance to vitamin D in breast cancer.

## 1. Introduction 

The insulin-like growth factor I (IGF-I) system is essential for normal growth and development. IGF-I is known to modulate control by insulin of normal carbohydrate and lipid metabolism. In addition, IGF-I has been reported to play a role in several pathological conditions. Interaction with the IGF binding proteins (IGFBPs) has been shown to both enhance and attenuate actions of IGF-I [1]. In addition, the IGFBPs are known to possess intrinsic growth regulatory activity, independent of their interactions with IGF-I. Insulin-like growth factor I (IGF-I) is implicated in breast cancer development and has been shown to rescue breast cancer cells from apoptosis induced by a range of chemotherapeutic agents [2]. Cellular responsiveness to IGF-I growth stimulation depends on the expression and activity of the signal transducing IGF-I receptor (IGF-IR) and a family of structurally related insulin-like growth factor binding proteins (IGFBP-1 to IGFBP-7). The major carrier of IGF-I in the circulation is IGFBP-3, which has been shown to inhibit cell growth and induce apoptosis in several cancer cell lines [3]. IGFBP-3 has been shown to regulate cell growth through both IGF-IR-dependent and -independent mechanisms (reviewed in [4]). The latter may involve signalling through an alternative cell surface receptor [5] or may involve direct nuclear actions by IGFBP-3 [6]. 

A number of factors with potent growth-inhibitory and apoptosis-inducing effects have been shown to induce the expression and secretion of IGFBP-3 in breast cancer cell lines, including 1, 25-dihydroxyvitamin D_3_ (1, 25-D_3_), the active metabolite of vitamin D_3_ which has been shown to inhibit breast cancer cell growth [7]. This finding suggests that IGFBP-3 may mediate or facilitate the inhibitory effects of 1, 25-D_3_. The aim of our study was to evaluate the role of  IGFBP-3 in 1, 25-D_3_-induced apoptosis in breast cancer cells. To this end, IGFBP-3 expression and secretion were investigated in parental MCF-7 breast cancer cells and the 1, 25-D_3_-resistant cell line MCF-7/VD^R^. This cell line is a vitamin-D-resistant clone of MCF-7 cells, which was developed by incubation of parental cells with a low concentration of 1, 25-D_3_, separating out the viable (resistant) cells and repeating this procedure with increasing concentrations of 1, 25-D_3_ [8]. This cell line contains fully functional VDR, although in a lower number than seen with the parental MCF-7 cells. The regulation of the 24-hydroxylase enzyme appeared to be intact and no differences with regard to growth rate and morphological appearance between parental and resistant clone were observed. The MCF-7/VD^R^ cell line thus provides a valuable tool for identifying the exact mechanism of action of vitamin D and the development of vitamin D resistance.

## 2. Materials and Methods

### 2.1. Cell Culture and Reagent

MCF-7 human breast cancer cells were obtained from the European tissue culture collection and used between passages 5 and 20. Vitamin-D-resistant MCF-7/VD^R^ cells were obtained as a gift from Dr. Mork Hansen [8]. Both parental and resistant cells were grown in RPMI 1640 supplemented with 2 mM of glutamine, 100 IU/mL of penicillin, 100 *μ*g/mL of streptomycin, and 2% of foetal bovine serum (FBS). 1, 25-D_3_ (Sigma UK) was used at a concentration of 100 nM and IGFBP-3 (R&D Systems) up to 100 nM. 

### 2.2. Viability Assay

MCF-7 and MCF-7/VD^R^ cells were seeded into 24 well plates at a density of 1 × 10^4^ cells/well. After 24 h, cells were treated with reagents or vehicle for up to six days. At the end of the incubation period, medium was removed and cells were incubated with neutral red solution (40 *μ*g/mL in phenol red-free medium) for 2 h at 37°C. Following washing, fixation, and solubilisation, absorbance at 550 nm was determined. 

### 2.3. Western Blot Analysis

Cells were lysed in radioimmunoprecipitation assay (RIPA) buffer containing 1% NP40, 0.5% sodium deoxycholate, 0.1% SDS, 1X PBS. Equal amounts of protein (30 *μ*g per lane) were subjected to SDS-PAGE and transferred to nitrocellulose membranes. Membranes were blocked with 5% milk in 0.05% Tween-20/TBS and then incubated with the primary antibody of interest overnight. Membranes were then incubated with the appropriate secondary horseradish-peroxidase-conjugated antibody. Bands were visualised using the enhanced chemiluminescence Western blotting detection system (ECL, Amersham). Anticleaved caspases 7, 8, and 9 and Poly [ADP-ribose] polymerase 1 (PARP-1) and antitotal caspases 7, 8, and 9, phospho-Akt, and PARP-1 antibodies were purchased from Cell Signalling. Anti-*β*-actin (Sigma Aldrich) was used as a loading control.

### 2.4. RNA Isolation and cDNA Synthesis

Total RNA from cells was extracted by using the PureLink RNA Mini-kit (Invitrogen). The quantity and the quality of RNA extracted was estimated by Nano-drop Spectrophotometer. For the reverse transcription, 2 *μ*g of RNA was resuspended in 10 *μ*L of nuclease free water with 2 *μ*L random hexamer (50 *μ*g) and was incubated at 70°C for 5 min. Then, the samples were resuspended with 13 *μ*L of Master Mix (5 *μ*L RT 5X Buffer, 2.5 *μ*L of dNTP 10 mM, 0.5 *μ*L Rnase OUT 40 U/*μ*L, 0.5 *μ*L of Reverse Transcriptase (MMLV, Promega), and 3.5 *μ*L of Nuclease Free Water). This mix was run for 1 h at 42°C, 5 min at 95°C, and 5 min at 4°C. The cDNA was stored at −20°C. 

### 2.5. RT-PCR Analysis of IGFBP-3 mRNA

The primers used to amplify IGFBP-3 and 28S rRNA were IGFBP-3 forward (GAAGGGCGACACTGCTTTTTC), IGFBP-3 reverse (CCAGCTCCAGGAAATGCTAG), 28S forward (GTTCACCCACTAATAGGGAAC), and 28S reverse (GGATTCTGACTTAGAGGCGTT). PCR was carried out in a total volume of 50 *μ*L containing 3 *μ*L of cDNA sample and 10 *μ*M sense and antisense primers. The RT-PCR exponential phase was determined in 28 to 33 cycles to allow quantitative comparisons. IGFBP-3 cDNA was amplified at 94°C for 2 minutes followed by 33 cycles at 94°C for 45 seconds, 63°C for 45 sec, and 72°C for 1 minute. 28S cDNA was amplified at 94°C for 2 minutes followed by 28 cycles at 94°C for 45 seconds, 58°C for 45 sec, and 72°C for 1 minute. Final extension was performed at 72°C for 5 min. Amplification products (8 *μ*L) were resolved in 2% agarose gel, stained with ethidium bromide, and visualized under UV light.

### 2.6. Detection of IGFBP-3 Secretion in Medium by ELISA Assay

IGFBP-3 protein level in each 200 *μ*L of medium and 100 *μ*g of cell extract was determined using a human IGFBP-3 ELISA kit (RayBioTech, USA) according to the manufacturer's protocol.

### 2.7. Antibody Specific Array

Mitogen-activated protein kinases (MAPK) protein phosphorylation was measured in each 300 *μ*g of cell extracts using Human Phospho-MAPK Array Kit according to the manufacturer (Proteome Profiler; R&D Systems). Briefly, antibody array membranes were incubated with protein lysates and then incubated with antibody array biotinylated antibody. Finally the membranes were incubated with streptavidin HRP-conjugated antibody. Immunoreactivity was visualized using a chemiluminescent substrate. Densitometric analysis was performed using GS-800 Calibrated Densitometer (Bio-Rad, UK).

### 2.8. Statistics

Data are reported as mean ± SD and analyzed with one-way ANOVA followed by the Bonferroni posttest for multiple comparisons using GraphPad Prism version 4.0. A value of *P* < 0.05 was considered significant. 

## 3. Results

### 3.1. Effects of 1, 25-D_3_ on Growth and IGFBP-3 Expression in Parental MCF-7 and Resistant MCF-7/VD^R^ Cells

**Figure 1:**
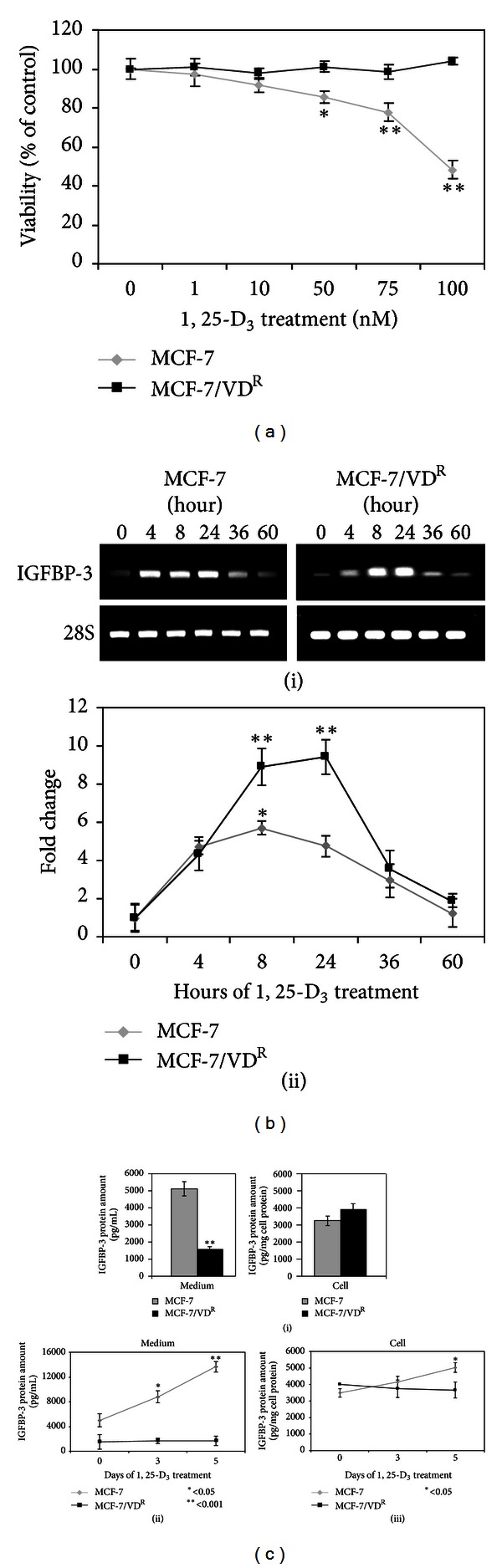
Effect of 1, 25-D_3_ on MCF-7 and MCF-7/VD^R^ cell viability and IGFBP-3 expression. (a) MCF-7 and MCF-7/VD^R^ cells were treated with increasing concentrations of 1, 25-D_3_ (up to 100 nM) or 0.1% ethanol vehicle as a control for 6 days. Cell viability was determined by neutral red assay. Means of 3 separate experiments are shown. *P < 0.05 and **P < 0.001 are statistically significant compared to the control. (b) (i) MCF-7 and MCF-7/VD^R^ cells were treated with 100 nM 1, 25-D_3_ for up to 60 hours. IGFBP-3 mRNA expression was measured by RT-PCR. 28S mRNA expression was used as house-keeping gene. Nontreated cells were used as controls. (ii) Densitometric analysis of of IGFBP-3 mRNA expression. Data shown means of three separate experiments. (c) Intracellular IGFBP-3 levels and secretion into medium was determined by ELISA in MCF-7 and MCF-7/VD^R^ cells. (i) IGFBP-3 expression and secretion into the medium in untreated MCF-7 and MCF-7/VD^R^ cells. (ii) IGFBP-3 secretion into the medium in MCF-7 and MCF-7/VD^R^ cells treated with 100 nM of 1, 25-D_3_ for up to 5 days quantitated by ELISA. (iii) The amount of intracellular IGFBP-3 production by MCF-7 and MCF-7/VD^R^ cells treated with 100 nM of 1, 25-D_3_ was measured at day 0, 3, and 5 by ELISA. Means of 3 separate experiments are shown. *P < 0.05 and **P < 0.001 are statistically significant compared to the control.

MCF-7 and MCF-7/VD^R^ cells were treated with increasing concentrations of 1, 25-D_3_ for up to 6 days. Cell viability was examined by neutral red dye assay (Figure 1(a)). Whilst 1, 25-D_3_ significantly decreased viability of MCF-7 cells, it had no significant effect on MCF-7/VD^R^ cell viability (Figure 1(a)). To determine effects on IGFBP-3 mRNA expression, MCF-7 and MCF-7/VD^R^ cells were treated with 100 nM 1, 25-D_3_ for up to 5 days. Whole RNA was extracted from the cells at different times of treatment and IGFBP-3 expression was examined by RT-PCR (Figure 1(b)). In both MCF-7 and MCF-7/VD^R^ cells, 1, 25-D_3_ treatment enhanced IGFBP-3 mRNA expression indicating that 1, 25-D_3_ was effective in inducing IGFBP-3 mRNA expression irrespective of the observed resistance in MCF-7/VD^R^ cells to 1, 25-D_3_-induced apoptosis (Figure 1(b)). 

Next, we examined effects of 1, 25-D_3_ on IGFBP-3 at the level of protein expression and secretion in MCF-7 versus MCF-7/VD^R^ cells. Cells were treated with 100 nM 1, 25-D_3_ for up to 5 days. The amount of IGFBP-3 protein present in the cell or secreted into the medium was assessed by ELISA. The basal level of intracellular IGFBP-3 protein expression was found to be similar in both cell lines (*P* > 0.05); however, the amount of IGFBP-3 protein in medium conditioned by parental MCF-7 cells was significantly higher than for the resistant cell line (*P* < 0.001), indicating a reduced secretion of IGFBP-3 by the MCF-7/VD^R^ cells (Figure 1(c)). In addition, 1, 25-D_3_ treatment induced IGFBP-3 protein expression and secretion in MCF-7 but not in MCF-7/VD^R^ cells (*P* < 0.05 and *P* < 0.001, resp.). Taken together, these results showed that impaired secretion but not transcriptional regulation of IGFBP-3 is associated with resistance of MCF-7/VD^R^ to 1, 25-D_3_. 

### 3.2. ****1, 25-D_3_- and IGFBP-3-Induced Apoptosis in MCF-7 and MCF-7/VD^R^ Cells

**Figure 2:**
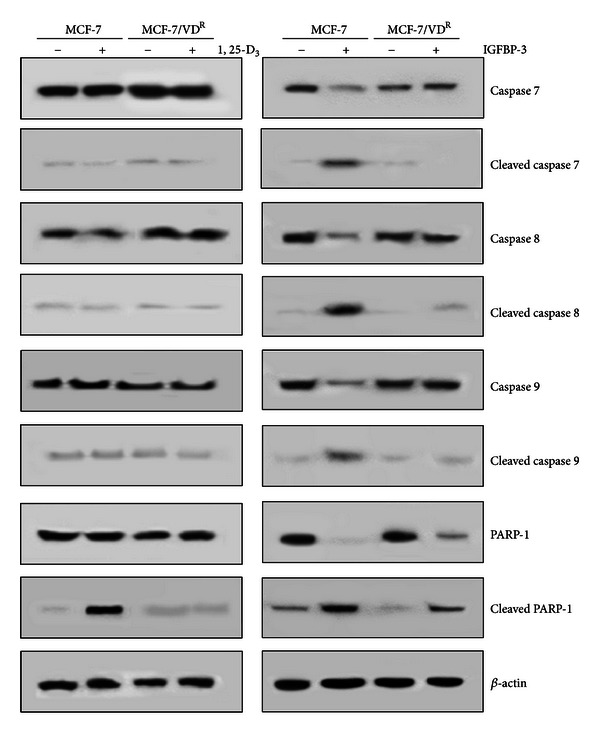
Caspase activation in response to 1, 25-D_3_ and IGFBP-3. MCF-7 and MCF-7/VD^R^ cells were treated with 100 nM 1, 25-D_3_ or 0.1% ethanol as vehicle control for 5 days (left hand panel) or 100 nM IGFBP-3 for 3 days. Control cells received an equal volume of PBS diluent (right-hand panel). Whole cell extracts were prepared and analysed by immunoblotting using specific antibodies of interest and *β*-actin was used as a loading control. Data shown are representative of three identical experiments.

To compare characteristics of 1, 25-D_3_- and IGFBP-3-induced apoptosis, parental and MCF-7/VD^R^ cells were treated for 5 days with 100 nM 1, 25-D_3_ or 100 nM IGFBP-3. Activation of caspases 7, 8, and 9 was monitored by detection of cleaved (active) caspase fragments by immunoblotting. In addition, PARP-1 cleavage was examined and *β*-actin was used as a house-keeping protein (Figure 2). 1, 25-D_3_ treatment did not lead to activation of any caspase but induced PARP-1 cleavage in parental MCF-7 but not in MCF-7/VD^R^ cells. In contrast, IGFBP-3 treatment produced cleavage of caspases 7, 8, and 9 and PARP-1 in MCF-7 cells. IGFPB-3 failed to activate caspases in MCF-7/VD^R^ cells; however PARP-1 cleavage was detected indicating an alternative pathway by which the protein induces apoptosis in these vitamin-D-resistant cells. 

### 3.3. Effect of 1, 25-D_3_ and IGFBP-3 on IGF-I/Akt Survival Signalling in MCF-7 and MCF-7/VD^R^ Cells

**Figure 3:**
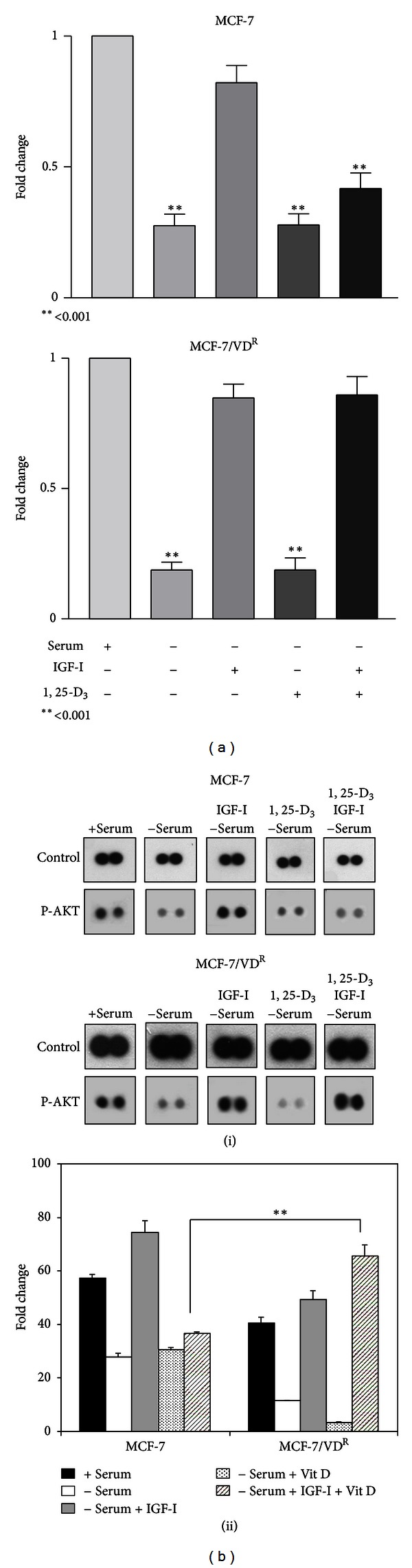
Modulation of IGF-induced Akt phosphorylation in response to 1, 25-D_3_ treatment in MCF-7 and MCF-7/VD^R^ cells. (a) MCF-7 and MCF-7/VD^R^ cells were treated with 100 nM 1, 25-D_3_ or 30 nM IGF-I, alone or in combination in serum-free medium. Cells were also cultured in medium supplemented with 2% serum as a control. After 6 days of treatment, cell viability was estimated by neutral red assay. *P < 0.05 and **P < 0.001 are statistically significant compared to control. Means of 3 separated experiments are shown (*n* = 12). (b) (i) MCF-7 and MCF-7/VD^R^ cells were treated with 100 nM 1, 25-D_3_ or 30 nM IGF-I, alone or in combination, in serum-free medium. After 5 days of treatment, whole cell extracts were prepared and analysed on a phospho-MAPK antibody array (R&D Systems, UK) following manufacturer's instruction. (ii) Densitometric analysis of Akt phosphorylation. Data shown are means of 3 replicates, significantly different from parental cells. **P < 0.001.

While parental MCF-7 cells do not express detectable IGF-I [9], the cells respond to the mitogenic and antiapoptotic effects of exogenous IGF-I and previous experiments have demonstrated that vitamin D treatment can attenuate the survival effect of IGF-I in parental MCF-7 cells [10]. To compare effects on IGF-I-mediated cell survival, MCF-7 and MCF-7/VD^R^ cells were treated with 100 nM 1, 25-D_3_ and 30 nM IGF-I, alone or in combination in serum-free medium and cell viability was examined by neutral red dye assay. Cells were also cultured in medium supplemented with 2% foetal bovine serum as a control. For both cell lines serum deprivation induced up to 70–80% of cell death compared to cells cultured in the presence of serum (*P* < 0.001) and addition of IGF-I to serum-free medium rescued cell viability (*P* > 0.05 compared to control). 1, 25-D_3_ treatment attenuated prosurvival effects of IGF-I in parental but not in resistant MCF-7/VD^R^ cells (Figure 3(a)). Failure of 1, 25-D_3_ to modulate IGF-I survival signalling in resistant cells could be due to differential regulation of IGF-I bioavailability by IGFBPs such as IGFBP-3, which is not secreted by these cells.

**Figure 4:**
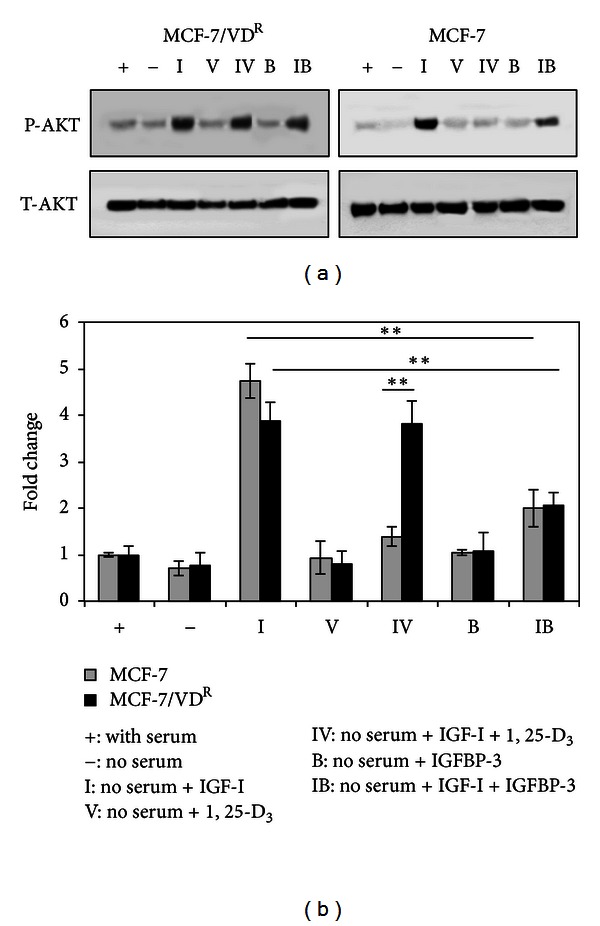
Differential modulation of IGF-induced Akt phosphorylation in response to 1, 25-D_3_ and IGFBP-3 treatment in MCF-7 and MCF-7/VD^R^ cells. (a) MCF-7 and MCF-7/VD^R^ cells were treated with 100 nM 1, 25-D_3_ or 30 nM IGF-I, alone or in combination, in serum-free medium. Cells were also treated with 100 nM IGFBP-3 alone or in combination with 30 nM IGF-I in serum-free medium. After 5 days of treatment, whole cell extracts were prepared and analysed by immunoblotting for total-Akt (T-Akt) and phospho-Akt (P-Akt). (b) Densitometric analysis of immunoblots was performed using GS-800 Calibrated Densitometer (Bio-Rad UK). Data shown are representative of three identical experiments. Means of 3 separated experiments are shown. *P < 0.05 and **P < 0.001 are statistically significant.

 We next compared 1, 25-D_3_ and IGFBP-3 treatment on MAPK and Akt activation in parental and resistant cells since it is well documented that IGF-I/MAPK and IGF-I/Akt signalling plays a crucial role in proliferation and survival of breast cancer cells. Cells were treated for 5 days with 100 nM 1, 25-D_3_ and 30 nM IGF-I, alone or in combination in serum-free medium. Cells were collected and isolated proteins were analysed on human phospho-MAPK antibody array. With respect to Akt phosphorylation, 1, 25-D_3_ attenuated the positive effect of IGF-I on activation of Akt in MCF-7 cells but failed to do so in MCF-7/VD^R^ cells (Figure 3(b)). No significant differences in activation of ERK, JNK, and p38 were detected between the two cell lines with these treatments (data not shown). Differential effects of 1, 25-D_3_ in parental and resistant cells on IGF-I-stimulated Akt activation were confirmed by immunoblotting. In contrast, treatment with IGFBP-3 reduced IGF-I-stimulated Akt phosphorylation in both cell lines (Figures 4(a) and 4(b)).

## 4. Discussion

The IGFBPs are secreted proteins, which bind to IGFs with high affinity. The IGFBP family has 7 distinct subgroups, IGFBP-1 through 7, and their production is tissue-type specific. Approximately 98% of IGF-1 is always bound to one of these binding proteins. The IGFBPs help to lengthen the halflife of circulating IGFs in all tissues and enhance or attenuate IGF signaling depending on their physiological context. IGFBP-3 is the most abundant of the family and accounts for 80% of all IGF binding [11]. IGFBP-3 is known to control IGF-I signalling leading to differential regulation of cell growth and apoptosis [12, 13]. A number of growth factors and hormones, including 1, 25-D_3_, have been shown to induce the expression of IGFBP-3 in breast cancer cell lines [7]. Comparative expression profiling of human IGFBP genes in different cancer cells demonstrated that IGFBP-1, -3 and -5 are primary 1, 25-D_3_ target genes [14]. In breast cancer, it was shown that 1, 25-D_3_ causes cyclical IGFBP-3 mRNA accumulation with a periodicity of 60 min [15]. Accordingly, VDR also showed cyclical ligand-dependent association with the chromatin regions of its VDREs. Interestingly, HDAC4 and HDAC6 proteins, which are upregulated in a cyclical fashion in response to 1, 25-D_3_, show cyclical VDR ligand-induced association with VDRE regions of the IGFBP-3 gene. Available evidence indicates that IGFBP-4 and 6 are not primary 1, 25-D_3_ target genes [14]. IGFBP-5 can colocalize with VDR in the nucleus and modulate vitamin D responses in osteoblasts [16]. IGFBP-6 has also been reported to interact with VDR in the bone [17].

Although there is an increasing body of evidence that 1, 25-D_3_ exerts potent regulatory effects on breast cancer cell growth, differentiation, and apoptosis [18], the mechanisms involved are not fully understood. Our results demonstrate that 1, 25-D_3_ treatment leads to an increase in IGFBP-3 mRNA in both sensitive and resistant MCF-7 cell lines, suggesting that the resistance to 1, 25-D_3_ is not due to impairment in IGFBP-3 gene expression at the mRNA level. This result was not surprising because the MCF-7/VD^R^ cells have been reported to express a functional Vitamin D receptor [8]. We next determined if there was a difference between sensitive and resistant cells at the level of IGFBP-3 protein. Whilst there was no difference in basal intracellular IGFBP-3 protein expression, the level of IGFBP-3 in conditioned medium from resistant cells was significantly lower than medium from parental MCF-7 cells. Furthermore, we detected a clear impairment of increased expression and secretion of this protein in MCF-7/VD^R^ cells in response to 1, 25-D_3_ treatment compared to parental cells, suggesting that effective secretion of this protein facilitates 1, 25-D_3_ responsiveness. A functional role of secreted versus nonsecreted IGFBP-3 is an interesting issue in the literature. One study demonstrated that nuclear translocation of IGFBP-3 and induction of apoptosis in parental MCF-7 cells require IGFBP-3 secretion and reuptake [19]. In contrast, Battacharyya and colleagues [20] reported that secreted and non-secreted IGFBP-3 may be functionally equivalent in induction of apoptosis in prostate cancer cells. Several studies have indicated that structural modifications such as glycosylation [21] and phosphorylation [22] can affect IGFBP-3 binding activity. However an ELISA approach, as used in our study, was unable to detect any such differences in secreted IGFBP-3 from MCF-7 cells.

It has been previously reported that 1, 25-D_3_ and IGFBP-3 induce MCF-7 cell death [23, 24]. Available evidence suggests differences in the characteristics of 1, 25-D_3_- and IGFBP-3-induced apoptosis in MCF-7 cells. It was previously reported that 1, 25-D_3_ induced apoptosis in a caspase-independent manner in MCF-7 cells [25] and this is confirmed in our present study. Whilst we found that 1, 25-D_3_ treatment did not induce activation of caspases 7, 8, and 9 in either MCF-7 or MCF-7/VD^R^ cell line, PARP-1 cleavage was detected in parental but not resistant cells. Indeed, it has previously been demonstrated that PARP-1 cleavage associated with 1, 25-D_3_-induced apoptosis could involve other proteinases such as calpains [26]. In contrast, we found that exogenous IGFBP-3-stimulated activation of caspases 7, 8 and 9 in parental MCF-7 but not in MCF-7/VD^R^ cells. However PARP-1 cleavage was detected in response to IGFBP-3 treatment in both cell lines suggesting that IGFBP-3 produces PARP-1 cleavage in a caspase-independent manner in MCF-7/VD^R^ cells. PARP-1 processing leading to activation of nucleases and DNA fragmentation appears as a key point in the execution phase of apoptosis. In support of our findings other reports have demonstrated PARP-1 processing in the absence of any caspase activation suggesting the role of other proteases in this process [27–29]. The ability of IGFBP-3 to induce apoptosis by both caspase-dependent and caspase-independent mechanisms suggests that this protein could act through two different signalling pathways. This observation also suggests that biochemical properties of endogenous and secreted IGFBP-3 may differ from exogenous protein.

 A number of studies have indicated IGF-I-dependent and -independent mechanisms by which IGFBP-3 induces apoptosis. By limiting IGF-I bioavailability, IGFBP-3 controls signal transduction through the IGF-I receptor, including survival signalling and induction of cell death. Exogenous IGFBP-3 also appears to exert IGF-I-independent effects, activating apoptosis via novel or death receptor pathways [9, 30, 31]. In contrast, other studies have shown that IGFBP-3 modulates RXR/Nur77 signalling in the nucleus, thereby inducing apoptosis in a mitochondria-dependent manner [32]. We found that RXR-*α* is expressed only in parental MCF-7 but not in MCF-7/VD^R^ cells (unpublished observations) suggesting that a RXR/Nur pathway does not exist in these cells and this is supported by absence of caspase 9 activation in response to IGFBP-3. 

**Figure 5:**
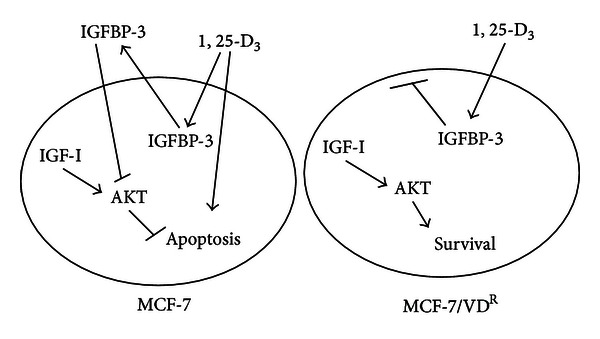
Proposed interaction between 1, 25-D_3_ and IGFBP-3 in MCF-7 and MCF-7/VD^R^ cells. In parental cells stimulation by 1, 25-D_3_ of IGFBP-3 secretion attenuates IGF-1-induced activation of Akt, leading to apoptosis. In addition, 1, 25-D_3_ may initiate caspase-independent pathways contributing to cell death in parental cells. In resistant cells, failure of IGFBP-3 secretion is associated with activation of the IGF-I/Akt pathway, leading to cell survival.

It is well documented that Akt activation plays a crucial role in antiapoptotic actions of IGF-I in breast cancer cells and our initial experiments clearly demonstrated that 1, 25-D_3_ treatment attenuated the survival effect of IGF-I in parental cell line but not in resistant MCF-7/VD^R^ cells. Our results using MAPK/Akt antibody array analysis demonstrated that 1, 25-D_3_ attenuated IGF-I-induced Akt phosphorylation in MCF-7 cells but failed to do so in MCF-7/VD^R^ cells, suggesting that failure to modulate IGF-I/Akt survival signalling could contribute to the resistance of this cell line to 1, 25-D_3_. In contrast, induction of apoptosis by exogenous IGFBP-3 in both 1, 25-D_3_-sensitive and -resistant cells was associated with inhibition the IGF-I/Akt pathway (Figure 5). In this regard the ability of IGFBP-3 to downregulate Akt activity in Her2 overexpressing MCF-7 cells has been previously reported [33]. 

## 5. Conclusion 

Taken together our results suggest a role for IGFBP-3 in 1, 25-D_3_ apoptotic signalling and impaired secretion of IGFBP-3 may be involved in acquired resistance to vitamin D in breast cancer cells. In addition, regulation of IGF-I/Akt survival signalling may function as a key point of convergence that may determine breast cancer cell fate in response to 1, 25-D_3_/IGFBP-3.
